# Assessment of harms, benefits, and cost‐effectiveness of prostate cancer screening: A micro‐simulation study of 230 scenarios

**DOI:** 10.1002/cam4.3395

**Published:** 2020-08-19

**Authors:** Abraham M. Getaneh, Eveline A. M. Heijnsdijk, Monique J. Roobol, Harry J. de Koning

**Affiliations:** ^1^ Department of Public Health Erasmus MC University Medical Center Rotterdam Rotterdam the Netherlands; ^2^ Department of Urology Erasmus MC University Medical Center Rotterdam Rotterdam the Netherlands

**Keywords:** harms and benefits; cost‐effectiveness, micro‐simulation, prostate cancer, prostate‐specific antigen (PSA) screening

## Abstract

**Background:**

Prostate cancer screening incurs a high risk of overdiagnosis and overtreatment. An organized and age‐targeted screening strategy may reduce the associated harms while retaining or enhancing the benefits.

**Methods:**

Using a micro‐simulation analysis (MISCAN) model, we assessed the harms, benefits, and cost‐effectiveness of 230 prostate‐specific antigen (PSA) screening strategies in a Dutch population. Screening strategies were varied by screening start age (50, 51, 52, 53, 54, and 55), stop age (51‐69), and intervals (1, 2, 3, 4, 8, and single test). Costs and effects of each screening strategy were compared with a no‐screening scenario.

**Results:**

The most optimum strategy would be screening with 3‐year intervals at ages 55–64 resulting in an incremental cost‐effectiveness ratio (ICER) of €19 733 per QALY. This strategy predicted a 27% prostate cancer mortality reduction and 28 life years gained (LYG) per 1000 men; 36% of screen‐detected men were overdiagnosed. Sensitivity analyses did not substantially alter the optimal screening strategy.

**Conclusions:**

PSA screening beyond age 64 is not cost‐effective and associated with a higher risk of overdiagnosis. Similarly, starting screening before age 55 is not a favored strategy based on our cost‐effectiveness analysis.

## Graphical abstract

Prostate cancer screening incurs a high risk of overdiagnosis and overtreatment. An organized and age‐targeted screening strategy may reduce the associated harms while retaining or enhancing the benefits. Screening men with 4 tests maximum, from ages 55 to 64, with 3‐year intervals is considered the optimum screening strategy.

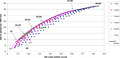

## 1 BACKGROUND

The incidence of prostate cancer has increased in most European countries, whereas prostate cancer mortality rates have declined.^1^, ^2^ Most Western European countries have experienced a sharp rise in the incidence of prostate cancer. The observed trend change in the incidence and mortality of prostate cancer may be partly related to opportunistic prostate‐specific antigen (PSA) screening and advances in prostate cancer treatment and diagnostic procedures.^3^ However, this progress is usually accompanied by a high risk of overdiagnosis. Various studies indicated that opportunistic PSA testing is less efficient and associated with a higher risk of overdiagnosis compared to organized screening.^4^, ^5^ An organized and age‐targeted screening strategy may reduce the associated harms while retaining or enhancing the benefits.

While screening for prostate cancer remains controversial, various large‐scale studies have confirmed the benefit of PSA screening.^6^, ^7^, ^8^ Similarly a secondary analysis confirmed that the Prostate, Lung, Colorectal and Ovarian screening Trial (PLCO) and European Randomized Study of Screening for Prostate Cancer (ERSPC) provide a compelling and consistent evidence that screening reduces prostate cancer mortality.^9^ However, the question as to the age at which PSA screening should start and especially at what age it should stop remains debatable, mainly because of the associated harms and costs. Finding the optimal screening strategy can lead to a better balance between the harms and benefits for citizens. Recently, the European Association of Urology (EAU) recommended that a baseline PSA test should be offered to men aged >50 and, men >45 years of age having a family history of prostate cancer or men of African‐American origin,^10^ whereas the US Preventive Services Task Force (USPSTF) recommended age 55 as the starting age and that the decision to undergo periodic PSA‐based screening for prostate cancer should be an individual one.^11^

Even though evidence for the benefit of prostate cancer screening under age 55 seems less conclusive, there are some studies that suggest a benefit of screening between ages 50 and 54. Recently, the 18‐year follow‐up study from the Goteborg randomized control trial, one center of the ERSPC trial, showed a large and statistically significant relative prostate cancer mortality reduction (RR = 0.31) for the attendees in this age group.^8^ Similarly, two other recent studies indicated a possible benefit of screening for this age group.^12^, ^13^ Although the overall result reported from the CAP (Cluster Randomized Trial of PSA Testing for Prostate Cancer) trial was insignificant, the highest prostate cancer mortality reduction was seen in this age group.^13^ The insignificant result from the CAP trial may be related to the single screening offered and its lower acceptance rate (36%).^14^

Although multiple studies on prostate cancer screening have been conducted, they have mainly focused on screening starting at age 55^6^, ^7^, ^15^, ^16^ or did not calculate life years gained or quality‐adjusted life years (QALYs) gained.^17^, ^18^, ^19^ Furthermore, finding an optimum screening strategy requires comparison of several screening strategies. The present study aimed to assess the harms, benefits, and an optimum cost‐effectiveness scenario of prostate cancer screening for men from age 50 onwards in a Dutch population. A total of 230 screening strategies were evaluated using a micro‐simulation analysis model.

## 2 MATERIALS AND METHODS

### 2.1 Model description

**Figure 1:**
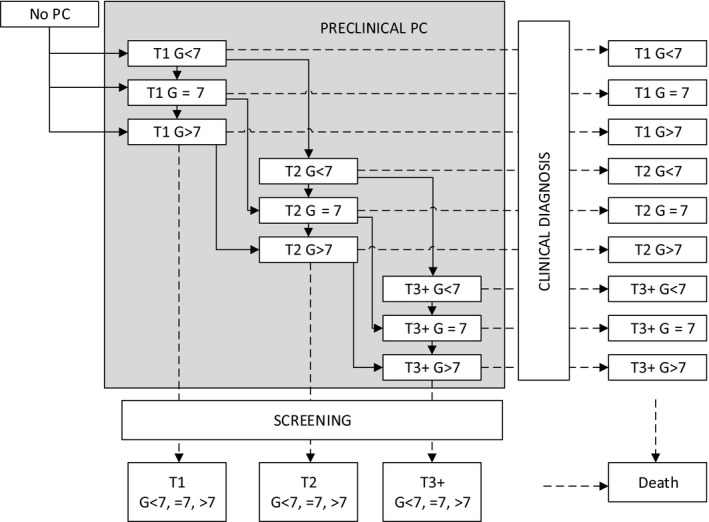
The MISCAN prostate cancer model. The model also contains a distinction between local and distant stages, but for the sake of simplicity it is not illustrated here. T, tumor stage; G, Gleason score

For this study we used a micro‐simulation screening analysis (MISCAN) model in order to assess the effects of prostate cancer screening. MISCAN prostate model has been described extensively before.^6^, ^20^ In short it is a stochastic model that simulates individual life histories of men and the natural life histories of prostate cancer. Overall, the model consists of 18 preclinical detectable states combined with three stages (T1, T2, and T3), three Gleason scores (7, <7, and >7) and two metastatic states (local‐regional and distant). Each individual in the simulation starts with no prostate cancer. Once the individual has prostate cancer, the cancer can progress to different screen‐detectable preclinical states. From each preclinical state, the cancer has a probability to progress to clinical prostate cancer (detected by symptoms) (Figure 1).

In the model, prostate cancer incidence and mortality are first simulated in the absence of screening. Prostate cancer survival in the absence of treatment (baseline survival) was estimated at clinical detection based on surveillance, epidemiology, and end results data from the pre‐PSA era (1983‐1986). Those clinically detected men with local disease and having received primary treatment (radical prostatectomy or radiation therapy) have improved survival rates with a hazard ratio of 0.56 compared to baseline survival.^21^ For distant cases it is assumed that treatment has no effect on survival. Following this, the effect of PSA screening on the natural history of prostate cancer is simulated. In our model, the effect of PSA screening on prostate cancer mortality is dependent on the lead time using a lead time‐dependent cure probability.^22^

In our model, the allocation of treatments (radical prostatectomy, radiation therapy, and active surveillance) after the diagnosis of prostate cancer was based on age, stage, and Gleason score as described in previous studies.^3^, ^22^ It was assumed that 30% of men switch from active surveillance to secondary treatment during the first 7 years.^6^ A Dutch life table was applied to model nonprostate cancer‐related death.^23^

### 2.2 Model calibration

The MISCAN prostate model was previously calibrated to ERSPC data by estimating parameters on duration, sensitivity, and lead time–dependent cure probability.^15^ In order to adapt the model to the Dutch situation and also account for younger age groups (50‐54), the model was calibrated to prostate cancer incidence among the Dutch population between 1989 and 2013 by 5‐year age categories from age 50 to 75.^24^ Furthermore, prostate cancer mortality predicted by the model was compared with observed prostate cancer mortality (among the Dutch population) over the same period (1989‐2013) to validate our model. More information on the calibration of the model is available in the supplementary part of this manuscript. Additional descriptions about the four components of MISCAN prostate model (demography, natural history, screening, and treatment) can be found at https://cisnet.flexkb.net/mp/pub/CISNET_ModelProfile_PROSTATE_ERASMUS_001_12152009_69754.pdf.

### 2.3 Screening strategies

A hypothetical cohort of 10 million men in the Netherlands aged 50 in 2020 was sampled and simulated over a lifetime period. The reason why we used a larger sample size than the male population in the Netherlands is to avoid a stochastic noise in the model. This number was selected by increasing the sample size until the model outputs get stable. Screening strategies were varied by screening start age, stop age, and screening intervals. The screening start age varied between 50 and 55 years, and the age at which screening was stopped varied between the screening start ages and age 69. Screening intervals of 1, 2, 3, 4, and 8 years and once‐in‐a‐lifetime screenings were applied.

In our study the biopsy compliance rate after a positive screen test result was assumed to be 90%, with a sensitivity of 90% as observed in the ERSPC Rotterdam data.^25^, ^26^ Most ERSPC centers used a PSA cutoff value of 3 ng/mL as an indication for biopsy,^27^ and a similar cutoff was used in our model. A screening attendance of 80% was assumed. For each strategy a total number of invitations, PSA tests done, prostate cancer detected (with and without screening), overdiagnosed cancer, prostate cancer death (with and without screening), and life years gained were predicted. The total number of biopsies was estimated by using the number of screen‐detected cancers and a mean positive predictive value of 22.7% of a biopsy in the screen arm of the ERSPC^26^ and by using the number of clinically detected cancers and the positive predictive value of 35.8% of a biopsy in the control arm.^28^

For each screening strategy, overdiagnosis was estimated as a proportion of screen‐detected prostate cancers (ie, overdiagnosed prostate cancers divide by screen‐detected prostate cancers). The screen‐detected prostate cancers composed of both overdiagnosed prostate cancers and relevant (nonoverdiagnosed) prostate cancers. The term overdiagnosis was defined as the detection of a prostate cancer during screening that would not have been clinically diagnosed during the man's lifetime in the absence of screening. All the outcomes (costs and effects) were estimated over a lifetime period and presented per 1000 men.

### 2.4 Quality of life, costs, and cost‐effectiveness

All utility estimates, unit costs (costs of screening, biopsy, primary treatment, follow‐up and palliative care for advanced cases), and durations in screening, biopsy, and treatment phases were obtained from a previous study^6^ (Table S1). Our analysis did not consider indirect costs. As described in a previous study,^15^ the utility estimates for the postrecovery period was obtained by combining the percentage of men with side effects from treatment with the utility estimates for those side effects. This resulted in utility estimates of 0.95 for all men during the period of 1‐10 years after diagnosis and after receiving radical prostatectomy or radiation therapy. The utility estimates range between 0 (death) and 1 (perfect health) and one minus the utility estimate gives a loss in utility at each health state. The total loss in quality of life was estimated as follows:

$$\sum_{i=1}^{k}\left(1‐u_{i}\right)\times d_{i}\times n_{i}$$

where u, d, and n represent the utility estimate, duration (in years, eg 2 months = 1/6 year), and number of men in each health state (i), respectively. The utility estimates and durations are presented in Table S1. The number of men in each health state was based on the model prediction. The letter “k” indicates the total number of health states.

QALYs gained were calculated by subtracting the total loss in quality of life from the net life years gained as a result of screening.

After determining the costs and effects of each screening strategy, the results were compared with a no‐screening scenario. Both strategies that were at least as expensive as and less effective (also called “strongly dominated strategies”) than an alternative option and weakly dominated strategies were excluded from the cost‐effectiveness analyses. A weakly dominated strategy is defined as a strategy whose incremental cost‐effectiveness ratio (ICER) is greater than that of a more effective strategy.^29^ The remaining strategies were regarded as efficient strategies and listed from lowest to highest according to their ICER. The ICER was calculated as the additional costs divided by additional QALYs gained compared with the previous less expensive strategy. The optimum efficient strategy was identified by comparing the ICERs with the willingness‐to‐pay (WTP) threshold per QALY. Considering a commonly used WTP threshold of €20 000 in a Dutch situation,^30^ a strategy (among efficient strategies) with the highest ICER below this threshold was taken as the optimum strategy. All costs and effects were estimated at a discount rate of 3.5% and presented in comparison with the no‐screen scenario, unless otherwise stated.

### 2.5 Sensitivity analysis

Univariate sensitivity analyses were conducted to test the robustness of the model results under different assumptions. Utility estimates of different health states and costs of screening, diagnosis, and treatment were the selected parameters for these analyses. The utility estimates in each health state (except for the terminal illness and palliative therapy) were varied using the highest (favorable) and lowest (unfavorable) value (Table S1). For the terminal illness and palliative therapy, it is favorable for screening when the utility is low.^15^ All costs were varied by ±20%.

## 3 RESULTS

### 3.1 Calibration and validation

Our model adequately predicted the prostate cancer incidence trends in the Netherlands between 1989 and 2013 (Figure S1). Furthermore, the model reasonably predicted the prostate cancer mortality in the Netherlands (except for the 70‐74 age group) over the same time period (1989‐2013), and this was taken as validation of the model (Figure S2).

### 3.2 Effects of various screening strategies

**Figure 2:**
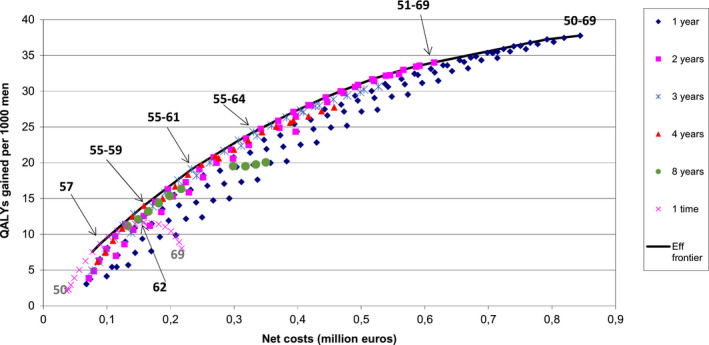
Net costs and QALYs gained per 1000 men. The start and end age of most optimal strategies given 1, 2, 3, 4, 8, and once depicted in the figure. Numbers in the legend indicate the screening intervals used in the model. Eff frontier, efficient frontier

For single screening strategies (once only), screening at age 57 was found to be most efficient which resulted in a 9.5 life years gain and 8.2% prostate cancer mortality reduction, with 31% of screen‐detected cancer overdiagnosed. Screening at 4‐year interval from age 55 to 59 (2 tests) and at 3‐year interval from age 55 to 61 (3 tests) were found to be other efficient strategies with ICER below the optimum cost‐effectiveness cutoff (Table 1). Screening at 3‐year intervals from age 55 to 64 (4 tests) was regarded as the optimum screening strategy with an ICER closest to the optimum cost‐effectiveness cutoff. Biennial screening between 51 and 69 (9 tests) and annual screening between age 50 and 69 (20 tests) were accompanied by a maximum life years gain of 41 and 47 years per 1000 men with a 42% and 47% life time prostate cancer mortality reduction, respectively. However, these benefits were accompanied by a higher risk of overdiagnosis (39% and 41%, respectively) (Table 1) and higher net costs for the corresponding life years or QALYs gained (Figure S3, and Figure 2) compared to other strategies. The fewest life years were gained with a single screening at age 50. In a one‐time screening strategy, the highest QALYs were attained at age 62. For all screening intervals used in our study, screening between an age group 50 and 54 generally yielded a lower number life years gain and prostate cancer mortality reduction than screening in age groups 55‐59 or 55‐64. The harms, benefits, and total net costs for each screening strategy are presented in the appendix (Table S2).

**Table 1:** Harms, benefits, and ICER for the efficient screening strategies. Results per 1000 men invited

Screening age	Number of tests	Screening interval	PCM reduction %	Overdiagnosis, as % of screen‐detected men	ICER in € Per QALY
56 single test	1	‐	6.9	29.3	10 211
57 single test	1	‐	8.2	30.7	10 946
55‐58	2	3	12.2	31.4	12 814
55‐59	2	4	13.8	31.6	13 129
55‐61	3	3	19.8	34.6	14 738
54‐63	4	3	25.1	34.7	18 417
55‐64	4	3	27.2	35.8	19 733
54‐64	6	2	30	34.9	22 395
55‐65	6	2	32.2	36	24 589
53‐65	7	2	33	35.6	24 819
54‐66	7	2	35	36.7	28 053
53‐67	8	2	37.6	37.4	29 565
52‐68	9	2	39	38.1	36 805
50‐68	10	2	40.3	37.9	43 831
51‐69	10	2	42	38.9	50 572
53‐69	17	1	46	38	55 083
52‐69	18	1	46.4	37.9	57 448
50‐69	20	1	46.9	41.3	97 784

Abbreviations: ICER, incremental cost‐effectiveness ratio; PCM, prostate cancer mortality; QALY, quality‐adjusted life years.

### 3.3 Cost‐effectiveness

The total costs of prostate cancer screening, diagnosis, and treatment ranged from €739 561 at no screening to €1 583 786 with annual screening of age 50‐69 per 1000 men (3.5% discounted). The ICER of efficient strategies, strategies on the efficient frontier, increased from €10 211 per QALY (single test at age 56) to €97 784 per QALY (annual screening between ages 50 and 69). As indicated in Table 1, most of the efficient strategies use a screening interval of 3 years or less, and screening strategies beyond age 64 were found to be less cost‐effective and associated with higher probabilities of overdiagnosis. Screening at 3‐year intervals from ages 55 to 64 resulted in an ICER of €19 733 per QALY, which is closest to the WTP threshold of €20 000 per QALY, and regarded as the optimum strategy. A 27% prostate cancer mortality reduction and 28 life years gained per 1000 men were predicated in association with this strategy. Of all screen‐detected men using this strategy, 36% were overdiagnosed. Extending the screening start age before age 55 (age 50 at the earliest) is less desirable (Table 1).

### 3.4 Sensitivity analyses

The results from the sensitivity analyses showed that for 77% of the analyses, screening from ages 55 to 64 with 3‐year screening intervals remained an optimal strategy, as in the base case scenario. Varying the utility estimate of the postrecovery period produced the greatest effect on screening stop age, screening frequency, and incremental cost‐effectiveness ratio of the optimum strategy. Using an unfavorable utility estimate for this parameter shifted the screening stop age of the optimum strategy from 64 to 61 (compared with the base case) with an ICER of €15 816. When the highest utility estimate was assumed for the same parameter, the screening stop age increased from 64 to 65, the screening frequency went from 3 to 2, QALYs gained rose from 24 to 33 (with a proportionate increase in the probability of overdiagnosis), and the ICER fell by 30%. A ±20% variation in unit costs caused the ICER of the optimum strategy to vary between €17 429 and €19 986 and also proportionately increased the effect of changing treatment costs (Table 2).

**Table 2:** Optimal strategies in base case and under a variety of different assumptions with their incremental cost‐effectiveness ratio

Parameter	Optimum strategy		ICER in €
	Screening age	Interval	
Base case	55‐64	3	19 733
Highest utility for screening attendance	54‐63	3	17 960
Lowest utility for screening attendance	55‐64	3	19 416
Highest utility for diagnostic phase	55‐64	3	19 371
Lowest utility for diagnostic phase	54‐63	3	18 582
Highest utility for diagnosis	55‐64	3	19 615
Lowest utility for diagnosis	55‐64	3	19 853
Highest utility at 2 mo after RP treatment	55‐64	3	19 284
Lowest utility at 2 mo after RP treatment	55‐64	3	19 956
Highest utility at 2 mo after RT treatment	55‐64	3	19 516
Lowest utility at 2 mo after RT treatment	55‐64	3	19 771
Highest utility at 2 mo to 1 y after RP treatment	55‐64	3	18 427
Lowest utility at 2 mo to 1 y after RP treatment	54‐63	3	19 427
Highest utility at 2 mo to 1 y after RT treatment	55‐64	3	18 835
Lowest utility at 2 mo to 1 y after RT treatment	54‐63	3	19 494
Highest utility for AS	55‐64	3	17 630
Lowest utility for AS	55‐61	3	19 217
Highest utility for postrecovery period	55‐65	2	19 150
Lowest utility for postrecovery period	55‐61	3	15 816
Highest utility for Palliative therapy	55‐61	3	17 085
Lowest utility for Palliative therapy	55‐67	2	18 133
Highest utility for terminal illness	54‐63	3	18 732
Lowest utility for terminal illness	55‐64	3	19 380
Costs of PSA test +20%	54‐63	3	18 710
Costs of PSA test −20%	55‐63	2	19 472
Costs of invitation +20%	55‐64	3	19 673
Costs of invitation −20%	55‐64	3	19 794
Costs of biopsy +20%	54‐63	3	18 664
Costs of biopsy −20%	55‐64	3	19 343
Costs of RP +20%	55‐64	3	19 562
Costs of RP −20%	55‐64	3	17 697
Costs of RT +20%	54‐63	3	19 986
Costs of RT −20%	55‐64	3	17 429
Costs of AS +20%	54‐63	3	18 710
Costs of AS −20%	55‐64	3	19 267
Costs of staging +20%	55‐64	3	19 815
Costs of staging −20%	55‐64	3	19 651
Costs of follow‐up +20%	55‐64	3	19 783
Costs of follow‐up −20%	55‐64	3	19 683
Costs of advanced case +20%	55‐64	3	18 940
Costs of advanced case −20%	54‐63	3	18 989

Abbreviations: AS, active surveillance, ICER, incremental cost‐effectiveness ratio, RP, radical prostatectomy; RT, radiation therapy.

## 4 DISCUSSION

According to the model predictions, the highest QALYs were estimated for age 62 in a one‐time screening strategy; extending once only screening to age 69 resulted in a loss in QALYs. However, extending the screening stop age yielded additional QALYs for the other strategies (Figure 2). This study shows that screening strategies with intervals of 4 years or shorter were more efficient than strategies with longer intervals. With 3‐year intervals, screening between ages 55 and 64 was found to be the optimum strategy. Screening beyond age 64 is less cost‐effective and associated with a higher risk of overdiagnosis.

When comparing screening between age group 50 and 54 and age groups 55 and 59 or 55 and 64, the former resulted in lower life years and QALYs gain, and lower prostate cancer mortality reduction than the other two age groups. The difference in prostate cancer mortality benefit between these strategies may be due to the lower chance of lethal prostate cancer among younger age groups.

An earlier study with our model showed an increasing trend in QALYs gained only up to age 63. QALYs started to fall when screening stop age extended beyond this age.^6^ A possible explanation for these contradictory results could be more effectiveness, and the lower overdiagnosis predicted in this study using the updated model compared to the previous one, because treating overdiagnosed cancer is the main cause of QALY loss. Updates in the model inputs (hazard of clinical prostate cancer detection and/or hazards of onset of a preclinical prostate tumor) in the current study could be the reason for the different overdiagnosis projections in the present and earlier study.^6^ On the other hand our findings are consistent with the earlier study with our model that screening is less cost‐effective at higher age and with longer screening intervals. When the optimum strategy in the current study was compared with that in the previous study (age group 55‐59 with 2‐year intervals), it resulted in 10 more life years gained at a much lower ICER and a 3% higher probability of overdiagnosis.^6^ The 27% prostate cancer mortality reduction estimated for the optimum strategy in the present study is in the same order as the 30% breast cancer mortality reduction reported in population‐based breast cancer screening, which is already established in the Netherlands.^31^

Generally, much lower net costs of screening and higher QALYs were predicted in the present study (Figure 2) as compared with some previous cost‐effectiveness studies.^6^, ^32^, ^33^ Factors that could explain this difference include differences in background risk (incidence), model assumptions, and proportions of cases assigned in each treatment category (radical prostatectomy, radiation therapy, and active surveillance). The higher QALYs gained reported in our study is in line with two previous studies.^15^, ^34^

Most of the results in our study are robust for the univariate sensitivity analyses. However, there are some parameters that produced a considerable effect on quality of life, which in turn altered the optimum strategy. Among these, the utility of postrecovery treatment is the principal one. This is due to the longer duration (9 years in our study) of this health state compared to the other health states. The use of a favorable utility estimate for this health state increased the QALYs gain by 8 at a lower ICER, whereas an unfavorable utility reduced the QALYs gain by 6 compared to the base case scenario. Men undergoing prostatectomy or radiation therapy for localized prostate cancer experience a decline in all functional outcomes (urinary, sexual, and bowel functions) throughout early, intermediate, and long‐term follow‐up.^35^

To our knowledge, the present study is the first that assesses the harms, benefits, and cost‐effectiveness of prostate cancer screening using Dutch population data. In addition, the existing studies, none of which are specific to the Netherlands, mainly focused on screening starting at age 55.^6^, ^7^, ^15^, ^16^ Therefore, the main strength of our study is that we were capable of considering screening before age 55, unlike several previous studies that mentioned this point as one of their study limitations.^6^, ^15^, ^16^ Another strength of this study is that we evaluated 230 screening scenarios, and find possible to recommend strategies when choosing for 1, 2, 3, or 4 tests.

Our study also had some limitations. Firstly, we did not use risk‐stratified screening. Several studies suggest risk‐based screening (for instance, screening based on PSA level) as one method to reduce overdiagnosis.^36^, ^37^ Similarly, various studies suggest that a magnetic resonance imaging (MRI)‐guided biopsy could minimize the risk of overdiagnosis,^38^, ^39^, ^40^ but MRI is not included in our screening protocol. We did not consider indirect costs in our analysis. Therefore, the actual total costs of prostate cancer screening may turn out to be higher than estimated in our study. Finally, our results are from a population‐based screening, and this may not be directly applicable in clinical practice under certain conditions. For instance, a man with high risk of prostate cancer may benefit from screening/rescreening beyond the screening stop age recommended in our study. Further studies that include selection of men based on their risk, such as using baseline PSA, comorbidity status, or using nomograms and/or MRI, as triage test may allow to screen older age groups with a minimal harm, or may improve the cost‐effectiveness.

In conclusion, our results indicate that PSA screening beyond age 64 is not cost‐effective and associated with a higher risk of overdiagnosis. Likewise, starting screening before age 55 is not a favored strategy based on our cost‐effectiveness analysis. Screening men with 4 tests maximum, from ages 55 to 64 with 3‐year intervals is considered the optimum screening strategy at a WTP threshold of €20 000.

## CONFLICT OF INTEREST

None.

## AUTHOR CONTRIBUTIONS

Conceptualization: All authors. Data curation: Abraham M. Getaneh and Eveline AM. Heijnsdijk. Formal analysis: Abraham M. Getaneh. Funding acquisition: Harry J. de. Koning. Investigation: Abraham M. Getaneh, Eveline AM. Heijnsdijk, and Harry J. de. Koning Methodology: Abraham M. Getaneh, Eveline AM. Heijnsdijk, and Harry J. de. Koning. Writing – original draft: Abraham M. Getaneh. Writing – review, and editing: All authors.

## Supporting information

Fig S1‐S3

Table S1‐S2

Supplementary Material

## Data Availability

The datasets used for calibration of the model for the current study are available in the Netherlands cancer registry (https://www.iknl.nl/nkr‐cijfers).
