# Supplementary material for: Assessment of harms, benefits, and cost‐effectiveness of prostate cancer screening: A micro‐simulation study of 230 scenarios
Source: Cancer Med. 2020 Aug 19;9(20):7742–50. doi: 10.1002/cam4.3395 (PMC7571827; doi:10.1002/cam4.3395)
Supplement: Supplementary file 1 — Fig S1‐S3 [file CAM4-9-7742-s001.pdf]

Appendix

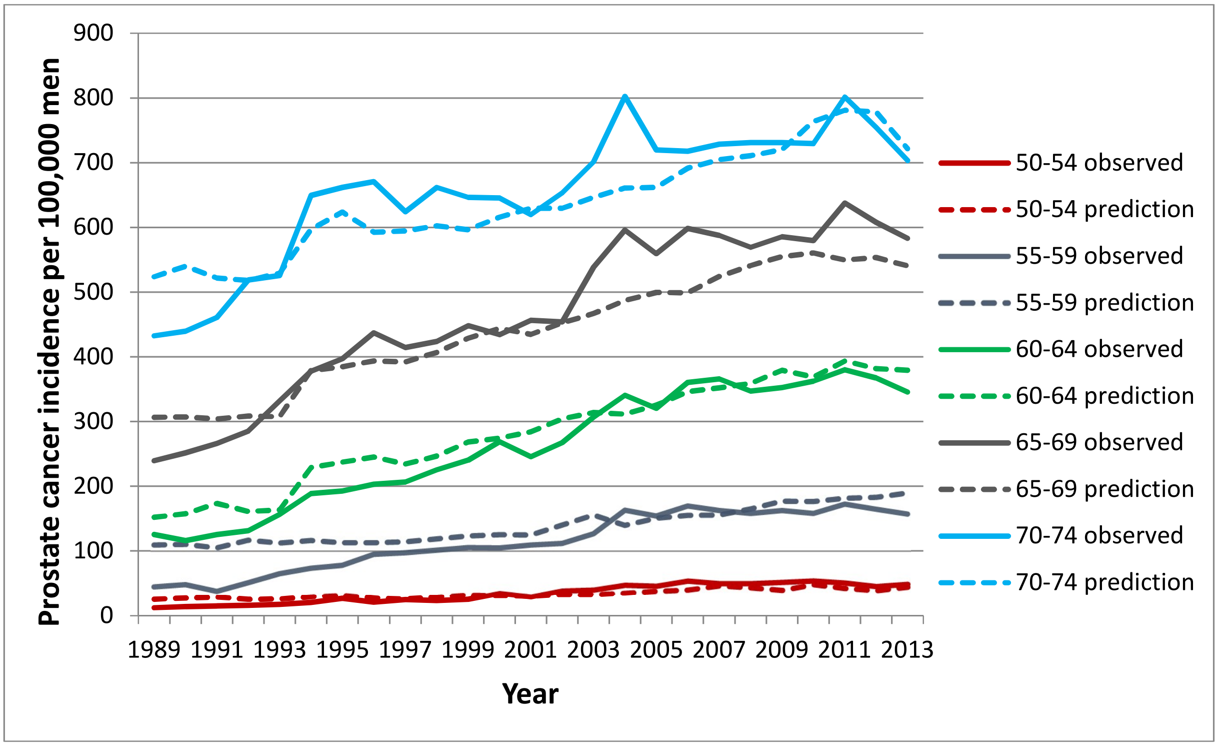

Appendix Figure 1. Observed and predicted prostate cancer incidence rates per 100,000 men across different age categories.

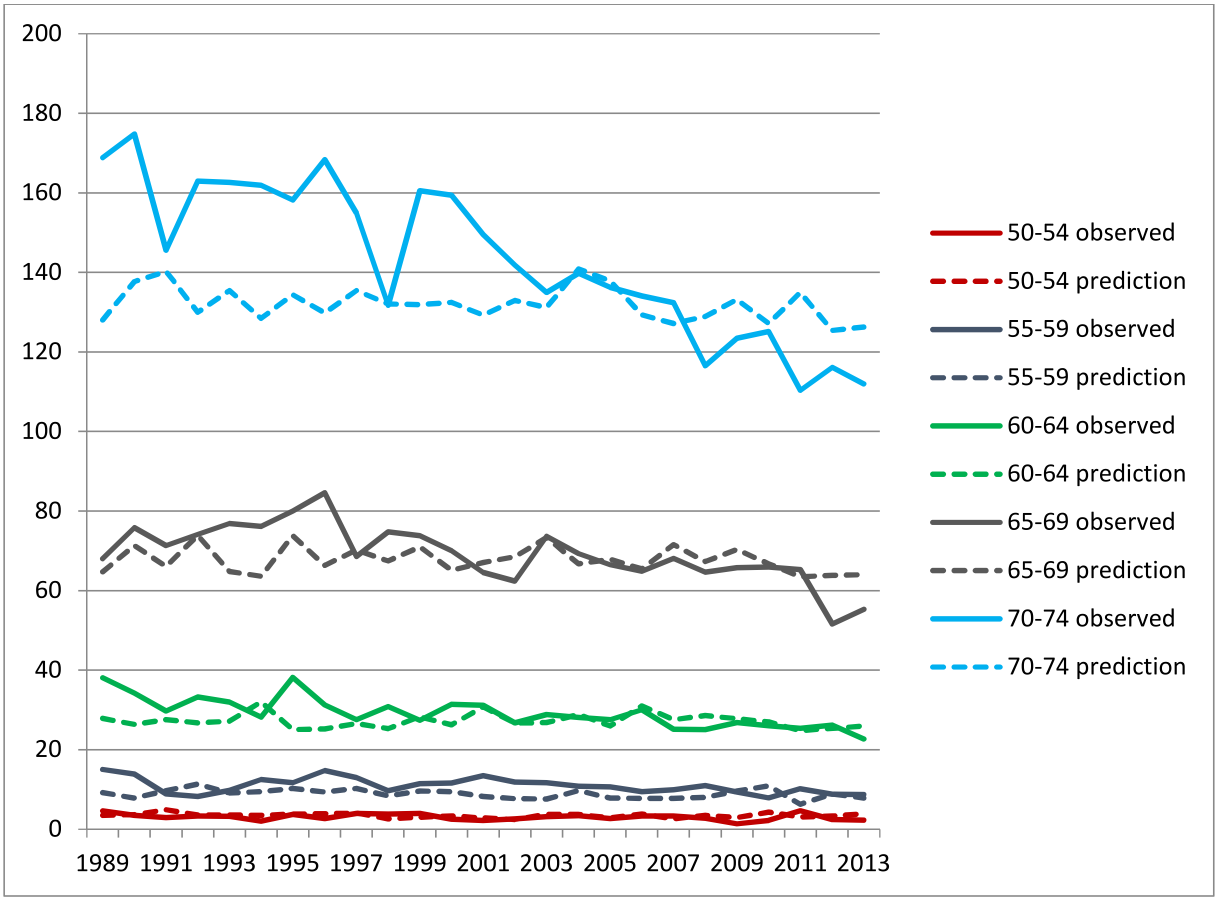

Appendix Figure 2. Observed and predicted prostate cancer mortality rates per 100,000 men across different age categories.

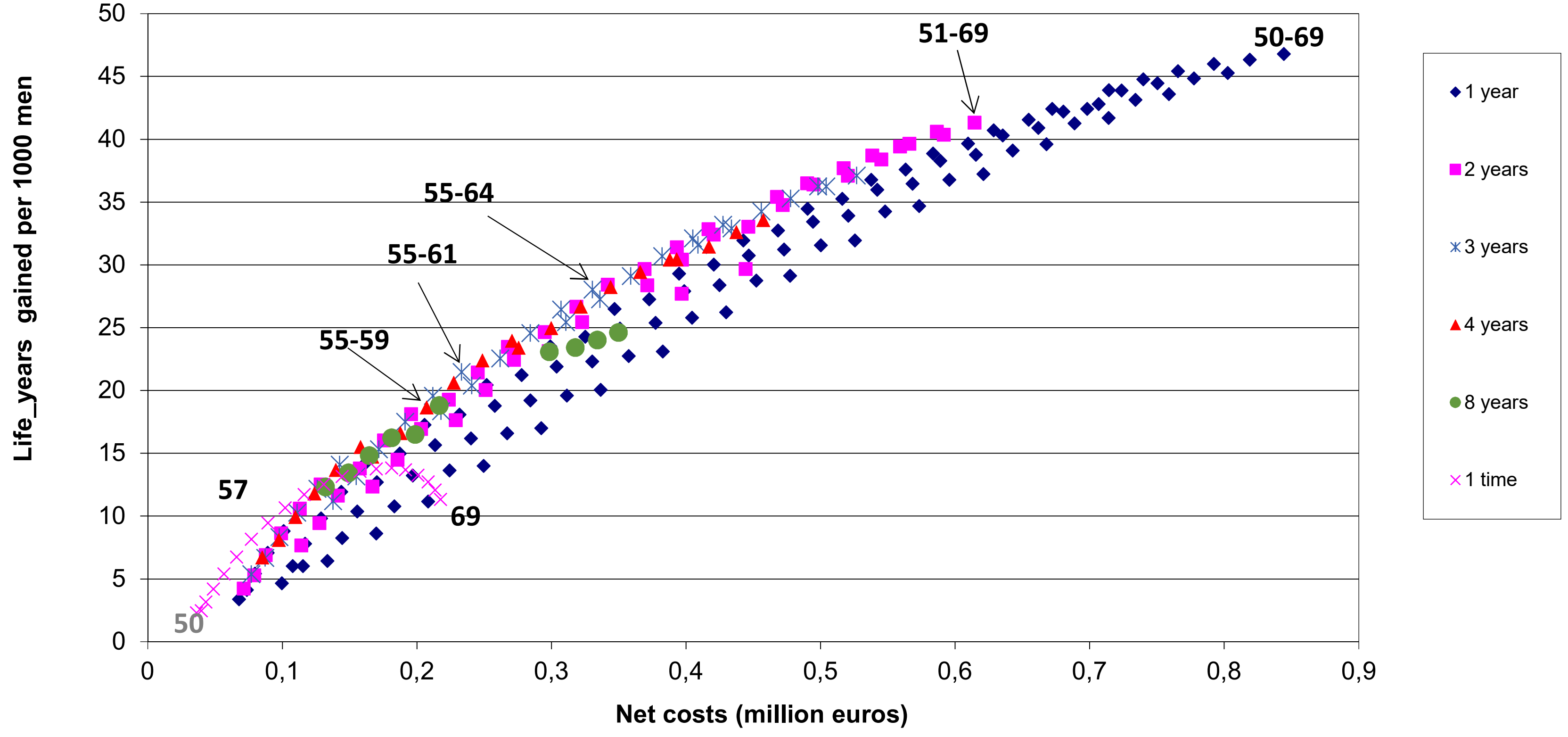

Appendix Figure 3. Net costs and life-years gained per 1000 men. The start and end age of most optimal strategies given 1,2,3,4,8 and once depicted in the figure. Numbers in the legend indicate the screening intervals used in the model.
