# Supplementary material for: Assessment of harms, benefits, and cost‐effectiveness of prostate cancer screening: A micro‐simulation study of 230 scenarios
Source: Cancer Med. 2020 Aug 19;9(20):7742–50. doi: 10.1002/cam4.3395 (PMC7571827; doi:10.1002/cam4.3395)
Supplement: Supplementary file 2 — Table S1‐S2 [file CAM4-9-7742-s002.docx]

Appendix Table 1. Costs, utility estimates and durations of the various phases in screening, diagnosis and treatment taken from a previous publication^6^ and costs were converted to euro. The utility estimate for the terminal illness was updated.

| Intervention | Unit costs in  euros | Health states | Utility estimates (range) | Duration |
| --- | --- | --- | --- | --- |
| Screening  Invitation  Blood sample taking  PSA determination | 34  2.8  13.5  17.7 | Screening attendance | 0.99 (0.98-1) | 1 week |
| Diagnosis  Biopsy  PA research  GP consulting | 241.3  130.7  47  63.6 | Diagnostic phase  Diagnosis | 0.90 ( 0.87- 0.94)  0.80 (0.75 -0.85) | 3 weeks  1 months |
| PT and follow up  Staging  RP  RT  AS  19 PSA tests  10 DRE  4 biopsies  Follow-up | 283.9  16,753.7  20,128.8  2,254.1  592.3  696.8  965  213.4 | RP  at 2 month after procedure  at >2 month to 1 y  RT  at 2 month after procedure  at >2 month to 1 y  Active surveillance  One year after treatment | 0.67 (0.56-0.90)  0.77 (0.70-0.91)  0.73 (0.71-0.91)  0.78 (0.61-0.88)  0.97 (0.85-1.00)  0.95 (0.93-1.00) | 2 months  10 months  2 months  10 months  Maximum 7 years  9 years |
| Advanced disease  Palliative therapy | 17,420 | Palliative therapy  Terminal illness | 0.60 (0.24-0.86)  0.40 (0.24-0.56 ) | 30 months  6 months |

DRE = digital rectal examination; GP = general practitioner; PA = pathological research; PSA = prostate-specific antigen; RP = radical prostatectomy; RT= radiation therapy; AS= Active surveillance (Active surveillance consists of multiple tests and corresponding costs are presented) .

Appendix Table 2. Harms, benefits and net costs of each screening strategies per 1000 menǂ

| **Screening age** | **Interval** | **LYs_gain#** | **PCMR %#** | Overdiagnosis, as % of screen detected men | **Total net costs #**  **in Euro** |
| --- | --- | --- | --- | --- | --- |
| 50-51 | 1 | 3.4 | 2.3 | 21.6 | 67,707 |
| 50-52 | 1 | 4.7 | 3.3 | 22.3 | 99,584 |
| 50-53 | 1 | 6.4 | 4.7 | 23.3 | 133,498 |
| 50-54 | 1 | 8.6 | 6.5 | 24.4 | 169,745 |
| 50-55 | 1 | 11.2 | 8.6 | 25.5 | 208,405 |
| 50-56 | 1 | 14 | 11.1 | 26.5 | 249,548 |
| 50-57 | 1 | 17 | 13.8 | 27.3 | 292,412 |
| 50-58 | 1 | 20 | 16.6 | 28.2 | 336,676 |
| 50-59 | 1 | 23.1 | 19.6 | 29.1 | 382,703 |
| 50-60 | 1 | 26.2 | 22.6 | 30 | 429,866 |
| 50-61 | 1 | 29.1 | 25.7 | 30.9 | 477,467 |
| 50-62 | 1 | 31.9 | 28.7 | 31.8 | 525,498 |
| 50-63 | 1 | 34.7 | 31.7 | 32.7 | 573,338 |
| 50-64 | 1 | 37.2 | 34.7 | 33.5 | 621,025 |
| 50-65 | 1 | 39.6 | 37.5 | 34.4 | 667,855 |
| 50-66 | 1 | 41.7 | 40.1 | 35.2 | 713,978 |
| 50-67 | 1 | 43.6 | 42.5 | 36.1 | 758,951 |
| 50-68 | 1 | 45.3 | 44.8 | 36.9 | 802,555 |
| 50-69 | 1 | 46.8 | 46.9 | 41.3 | 844,225 |
|  |  |  |  |  |  |
| 51-52 | 1 | 4.1 | 3 | 23 | 73,544 |
| 51-53 | 1 | 6 | 4.5 | 23.8 | 107,807 |
| 51-54 | 1 | 8.3 | 6.3 | 24.7 | 144,430 |
| 51-55 | 1 | 10.8 | 8.4 | 25.8 | 183,186 |
| 51-56 | 1 | 13.6 | 10.9 | 26.6 | 224,257 |
| 51-57 | 1 | 16.6 | 13.6 | 27.5 | 267,187 |
| 51-58 | 1 | 19.6 | 16.4 | 28.3 | 311,373 |
| 51-59 | 1 | 22.7 | 19.4 | 29.2 | 357,388 |
| 51-60 | 1 | 25.8 | 22.4 | 30.1 | 404,547 |
| 51-61 | 1 | 28.8 | 25.5 | 31 | 452,259 |
| 51-62 | 1 | 31.5 | 28.5 | 31.9 | 500,233 |
| 51-63 | 1 | 34.3 | 31.5 | 32.8 | 548,052 |
| 51-64 | 1 | 36.8 | 34.4 | 33.6 | 595,816 |
| 51-65 | 1 | 39.1 | 37.2 | 34.5 | 642,755 |
| 51-66 | 1 | 41.3 | 39.8 | 35.3 | 688,839 |
| 51-67 | 1 | 43.1 | 42.2 | 36.2 | 733,941 |
| 51-68 | 1 | 44.8 | 44.5 | 37 | 777,471 |
| 51-69 | 1 | 46.3 | 46.6 | 37.8 | 819,072 |
|  | 1 |  |  |  |  |
| 52-53 | 1 | 5.4 | 4.1 | 24.4 | 79,699 |
| 52-54 | 1 | 7.8 | 6 | 25.2 | 116,875 |
| 52-55 | 1 | 10.4 | 8.2 | 26.1 | 155,743 |
| 52-56 | 1 | 13.2 | 10.7 | 26.9 | 196,943 |
| 52-57 | 1 | 16.2 | 13.3 | 27.7 | 240,124 |
| 52-58 | 1 | 19.2 | 16.1 | 28.5 | 284,281 |
| 52-59 | 1 | 22.3 | 19.2 | 29.4 | 330,255 |
| 52-60 | 1 | 25.4 | 22.2 | 30.3 | 377,417 |
| 52-61 | 1 | 28.4 | 25.2 | 31.1 | 424,863 |
| 52-62 | 1 | 31.2 | 28.3 | 32 | 472,914 |
| 52-63 | 1 | 33.9 | 31.3 | 32.8 | 520,708 |
| 52-64 | 1 | 36.5 | 34.2 | 33.7 | 568,380 |
| 52-65 | 1 | 38.8 | 37 | 34.5 | 615,482 |
| 52-66 | 1 | 40.9 | 39.6 | 35.4 | 661,786 |
| 52-67 | 1 | 42.8 | 42 | 36.2 | 706,785 |
| 52-68 | 1 | 44.5 | 44.2 | 37.1 | 750,387 |
| 52-69 | 1 | 46 | 46.4 | 37.9 | 792,212 |
|  |  |  |  |  |  |
| 53-54 | 1 | 7.1 | 5.5 | 25.8 | 89,035 |
| 53-55 | 1 | 9.8 | 7.8 | 26.6 | 128,741 |
| 53-56 | 1 | 12.7 | 10.3 | 27.4 | 170,345 |
| 53-57 | 1 | 15.7 | 13 | 28.1 | 213,459 |
| 53-58 | 1 | 18.8 | 15.9 | 28.7 | 257,824 |
| 53-59 | 1 | 21.8 | 18.9 | 29.6 | 303,889 |
| 53-60 | 1 | 24.9 | 21.9 | 30.5 | 350,978 |
| 53-61 | 1 | 27.9 | 24.9 | 31.3 | 398,585 |
| 53-62 | 1 | 30.8 | 28 | 32.2 | 446,605 |
| 53-63 | 1 | 33.4 | 31 | 33 | 494,465 |
| 53-64 | 1 | 36 | 33.9 | 33.8 | 542,079 |
| 53-65 | 1 | 38.3 | 36.6 | 34.7 | 589,078 |
| 53-66 | 1 | 40.3 | 39.2 | 35.5 | 635,337 |
| 53-67 | 1 | 42.2 | 41.6 | 36.4 | 680,448 |
| 53-68 | 1 | 43.9 | 43.8 | 37.2 | 723,806 |
| 53-69 | 1 | 45.4 | 45.9 | 38 | 765,672 |
|  |  |  |  |  |  |
| 54-55 | 1 | 8.8 | 7.2 | 27.3 | 100,959 |
| 54-56 | 1 | 11.9 | 9.9 | 27.8 | 143,724 |
| 54-57 | 1 | 15 | 12.5 | 28.5 | 187,272 |
| 54-58 | 1 | 18.1 | 15.4 | 29.2 | 231,611 |
| 54-59 | 1 | 21.2 | 18.5 | 29.9 | 277,808 |
| 54-60 | 1 | 24.3 | 21.5 | 30.8 | 325,116 |
| 54-61 | 1 | 27.3 | 24.6 | 31.6 | 372,670 |
| 54-62 | 1 | 30 | 27.6 | 32.4 | 420,723 |
| 54-63 | 1 | 32.7 | 30.5 | 33.2 | 468,495 |
| 54-64 | 1 | 35.3 | 33.4 | 34.1 | 516,234 |
| 54-65 | 1 | 37.6 | 36.2 | 34.9 | 563,323 |
| 54-66 | 1 | 39.7 | 38.8 | 35.7 | 609,601 |
| 54-67 | 1 | 41.5 | 41.2 | 36.5 | 654,668 |
| 54-68 | 1 | 43.2 | 43.5 | 37.3 | 698,195 |
| 54-69 | 1 | 44.8 | 45.6 | 38.1 | 739,779 |
|  |  |  |  |  |  |
| 55-56 | 1 | 10.8 | 9 | 28.6 | 115,304 |
| 55-57 | 1 | 14.1 | 12 | 29.1 | 160,657 |
| 55-58 | 1 | 17.3 | 14.9 | 29.7 | 205,699 |
| 55-59 | 1 | 20.4 | 17.9 | 30.4 | 251,798 |
| 55-60 | 1 | 23.5 | 21 | 31.2 | 299,239 |
| 55-61 | 1 | 26.5 | 24.1 | 32 | 347,007 |
| 55-62 | 1 | 29.3 | 17.1 | 32.7 | 394,898 |
| 55-63 | 1 | 31.9 | 30 | 33.5 | 442,567 |
| 55-64 | 1 | 34.5 | 32.9 | 34.3 | 490,374 |
| 55-65 | 1 | 36.8 | 25.7 | 35.2 | 537,560 |
| 55-66 | 1 | 38.9 | 38.3 | 36 | 583,689 |
| 55-67 | 1 | 40.7 | 40.7 | 36.8 | 628,750 |
| 55-68 | 1 | 42.4 | 42.9 | 37.6 | 672,170 |
| 55-69 | 1 | 43.9 | 45 | 38.4 | 714,210 |
|  |  |  |  |  |  |
| 50-52 | 2 | 4.2 | 3 | 22.7 | 71,321 |
| 50-54 | 2 | 7.7 | 5.8 | 24.7 | 114,180 |
| 50-56 | 2 | 12.3 | 9.8 | 26.8 | 167,204 |
| 50-58 | 2 | 17.6 | 14.7 | 28.7 | 228,879 |
| 50-60 | 2 | 23.1 | 20.2 | 30.8 | 298,127 |
| 50-62 | 2 | 28.4 | 25.7 | 32.6 | 371,283 |
| 50-64 | 2 | 33 | 31.1 | 34.4 | 446,379 |
| 50-66 | 2 | 37.1 | 36 | 36.2 | 520,377 |
| 50-68 | 2 | 40.3 | 40.3 | 37.9 | 591,728 |
|  |  |  |  |  |  |
| 51-53 | 2 | 5.3 | 4 | 24.1 | 79,266 |
| 51-55 | 2 | 9.4 | 7.4 | 26.2 | 127,589 |
| 51-57 | 2 | 14.5 | 11.9 | 28.1 | 185,639 |
| 51-59 | 2 | 20 | 17.2 | 29.9 | 251,177 |
| 51-61 | 2 | 25.4 | 22.7 | 31.8 | 322,930 |
| 51-63 | 2 | 30.4 | 28.2 | 33.6 | 397,136 |
| 51-65 | 2 | 34.8 | 33.4 | 35.4 | 471,937 |
| 51-67 | 2 | 38.4 | 38 | 37.2 | 545,195 |
| 51-69 | 2 | 41.3 | 42 | 38.9 | 614,461 |
|  |  |  |  |  |  |
| 52-54 | 2 | 6.9 | 5.3 | 25.5 | 87,856 |
| 52-56 | 2 | 11.6 | 9.4 | 27.2 | 141,295 |
| 52-58 | 2 | 16.9 | 14.3 | 29.1 | 203,241 |
| 52-60 | 2 | 22.4 | 19.8 | 31 | 272,344 |
| 52-62 | 2 | 27.7 | 25.3 | 32.8 | 396,875 |
| 52-64 | 2 | 32.4 | 30.7 | 34.6 | 420,685 |
| 52-66 | 2 | 36.4 | 35.6 | 36.4 | 494,786 |
| 52-68 | 2 | 39.6 | 39 | 38.1 | 566,150 |
|  |  |  |  |  |  |
| 53-55 | 2 | 8.6 | 6.9 | 27 | 99,182 |
| 53-57 | 2 | 13.8 | 11.5 | 28.5 | 157,624 |
| 53-59 | 2 | 19.3 | 16.7 | 30.3 | 223,847 |
| 53-61 | 2 | 24.7 | 22.3 | 32.1 | 295,114 |
| 53-63 | 2 | 29.7 | 27.8 | 33.8 | 369,373 |
| 53-65 | 2 | 34 | 33 | 35.6 | 444,315 |
| 53-67 | 2 | 37.7 | 37.6 | 37.4 | 517,344 |
| 53-69 | 2 | 40.6 | 41.6 | 39.1 | 586,440 |
|  |  |  |  |  |  |
| 54-56 | 2 | 10.6 | 8.7 | 28.1 | 113,032 |
| 54-58 | 2 | 16 | 13.8 | 29.5 | 175,548 |
| 54-60 | 2 | 21.4 | 19.1 | 31.5 | 245,277 |
| 54-62 | 2 | 26.7 | 24.7 | 33.2 | 318,595 |
| 54-64 | 2 | 31.4 | 30 | 34.9 | 393,306 |
| 54-66 | 2 | 35.4 | 35 | 36.6 | 467,617 |
| 54-68 | 2 | 38.7 | 39.3 | 38.3 | 538,613 |
|  |  |  |  |  |  |
| 55-57 | 2 | 12.5 | 10.6 | 29.4 | 128,653 |
| 55-59 | 2 | 18.1 | 16 | 30.9 | 195,731 |
| 55-61 | 2 | 23.5 | 21.5 | 32.6 | 267,681 |
| 55-63 | 2 | 28.4 | 27 | 34.3 | 342,055 |
| 55-65 | 2 | 32.8 | 32.2 | 36 | 416,909 |
| 55-67 | 2 | 36.5 | 36.8 | 37.7 | 490,133 |
| 55-69 | 2 | 39.4 | 40.9 | 39.4 | 559,212 |
|  |  |  |  |  |  |
| 50-53 | 3 | 5.4 | 4 | 23.9 | 77,098 |
| 50-56 | 3 | 11.2 | 9 | 27.3 | 137,770 |
| 50-59 | 3 | 18.3 | 15.8 | 30.3 | 217,938 |
| 50-62 | 3 | 25.4 | 23.3 | 33.3 | 310,874 |
| 50-65 | 3 | 31.6 | 30.5 | 36.1 | 408,890 |
| 50-68 | 3 | 36.2 | 36.7 | 38.8 | 504,367 |
|  |  |  |  |  |  |
| 51-54 | 3 | 6.7 | 5.1 | 25.4 | 87,369 |
| 51-57 | 3 | 13.2 | 10.9 | 28.4 | 155,053 |
| 51-60 | 3 | 20.4 | 18 | 31.6 | 240,855 |
| 51-63 | 3 | 27.3 | 25.6 | 34.3 | 336,143 |
| 51-66 | 3 | 32.9 | 32.5 | 37.1 | 433,983 |
| 51-69 | 3 | 37.1 | 38.2 | 39.7 | 526,931 |
|  |  |  |  |  |  |
| 52-55 | 3 | 8.3 | 6.6 | 26.9 | 97,903 |
| 52-58 | 3 | 15.3 | 13.1 | 29.5 | 171,907 |
| 52-61 | 3 | 22.6 | 20.4 | 32.5 | 261,920 |
| 52-64 | 3 | 29.1 | 27.9 | 35.3 | 358,897 |
| 52-67 | 3 | 34.3 | 34.4 | 38.1 | 456,083 |
|  |  |  |  |  |  |
| 53-56 | 3 | 10.3 | 8.4 | 27.9 | 111,058 |
| 53-59 | 3 | 17.5 | 15.3 | 30.7 | 191,329 |
| 53-62 | 3 | 24.6 | 22.8 | 33.6 | 284,457 |
| 53-65 | 3 | 30.7 | 30 | 36.3 | 382,326 |
| 53-68 | 3 | 35.3 | 36.1 | 39.1 | 477,848 |
|  |  |  |  |  |  |
| 54-57 | 3 | 12.2 | 10.3 | 29.1 | 126,061 |
| 54-60 | 3 | 19.6 | 17.5 | 31.9 | 211,981 |
| 54-63 | 3 | 26.4 | 25.1 | 34.7 | 307,322 |
| 54-66 | 3 | 32.1 | 32 | 37.4 | 405,265 |
| 54-69 | 3 | 36.2 | 37.6 | 40 | 498,236 |
|  |  |  |  |  |  |
| 55-58 | 3 | 14.1 | 12.2 | 31.4 | 142,724 |
| 55-61 | 3 | 21.5 | 19.8 | 34.6 | 233,213 |
| 55-64 | 3 | 28 | 27.2 | 35.8 | 330,487 |
| 55-67 | 3 | 33.2 | 33.7 | 38.5 | 427,619 |
|  |  |  |  |  |  |
| 50-54 | 4 | 6.7 | 5.1 | 25.4 | 85,165 |
| 50-58 | 4 | 14.7 | 12.5 | 29.7 | 166,997 |
| 50-62 | 4 | 23.4 | 21.6 | 33.8 | 275,677 |
| 50-66 | 4 | 30.4 | 30.1 | 37.7 | 393,013 |
|  |  |  |  |  |  |
| 51-55 | 4 | 8.1 | 6.4 | 27 | 97,337 |
| 51-59 | 4 | 16.6 | 14.5 | 30.9 | 187,638 |
| 51-63 | 4 | 25 | 23.6 | 34.9 | 299,786 |
| 51-67 | 4 | 31.4 | 31.8 | 38.7 | 417,006 |
|  |  |  |  |  |  |
| 52-56 | 4 | 9.9 | 8.1 | 28 | 109,627 |
| 52-60 | 4 | 18.6 | 16.7 | 32.1 | 207,207 |
| 52-64 | 4 | 26.7 | 25.7 | 36 | 321,823 |
| 52-68 | 4 | 32.6 | 33.5 | 39.7 | 437,450 |
|  |  |  |  |  |  |
| 53-57 | 4 | 11.8 | 9.9 | 29.2 | 123,846 |
| 53-61 | 4 | 20.6 | 18.9 | 33.1 | 227,416 |
| 53-65 | 4 | 28.2 | 27.8 | 37 | 344,003 |
| 53-69 | 4 | 33.5 | 35 | 40.6 | 457,420 |
|  |  |  |  |  |  |
| 54-58 | 4 | 13.7 | 11.8 | 30.3 | 139,828 |
| 54-62 | 4 | 22.4 | 21 | 34.2 | 248,612 |
| 54-66 | 4 | 29.4 | 19.6 | 38 | 365,978 |
|  |  |  |  |  |  |
| 55-59 | 4 | 15.5 | 13.8 | 31.6 | 158,123 |
| 55-63 | 4 | 24 | 23 | 35.3 | 270,662 |
| 55-67 | 4 | 30.4 | 31.2 | 39.1 | 388,003 |
|  |  |  |  |  |  |
| 50-58 | 8 | 12.3 | 10.7 | 31 | 132,214 |
| 50-66 | 8 | 23.1 | 23.6 | 39.7 | 298,455 |
|  |  |  |  |  |  |
| 51-59 | 8 | 13.5 | 12.1 | 32.4 | 149,097 |
| 51-67 | 8 | 23.4 | 24.5 | 40.9 | 317,826 |
|  |  |  |  |  |  |
| 52-60 | 8 | 14.8 | 13.7 | 33.8 | 164,767 |
| 52-68 | 8 | 24 | 25.6 | 41.9 | 334,029 |
|  |  |  |  |  |  |
| 53-61 | 8 | 16.2 | 15.3 | 34.9 | 181,253 |
| 53-69 | 8 | 24.6 | 26.6 | 42.9 | 349,793 |
|  |  |  |  |  |  |
| 54-62 | 8 | 17.5 | 16.9 | 36 | 198,825 |
| 55-63 | 8 | 18.8 | 18.6 | 37 | 216,573 |
|  |  |  |  |  |  |
| 50 single test | - | 2.3 | 1.6 | 20.8 | 36,229 |
| 51 single test | - | 2.5 | 1.7 | 22.4 | 39,781 |
| 52 single test | - | 3.2 | 2.3 | 24 | 43,183 |
| 53 single test | - | 4.2 | 3.2 | 25.3 | 48,765 |
| 54 single test | - | 5.4 | 4.3 | 26.6 | 56,535 |
| 55 single test | - | 6.7 | 5.5 | 28.2 | 66,131 |
| 56 single test | - | 8.2 | 6.9 | 29.3 | 77,082 |
| 57 single test | - | 9.5 | 8.2 | 30.7 | 89,394 |
| 58 single test | - | 10.7 | 9.6 | 32 | 102,211 |
| 59 single test | - | 11.7 | 10.9 | 33.6 | 116,231 |
| 60 single test | - | 12.5 | 12 | 35.3 | 130,803 |
| 61 single test | - | 13.2 | 13.1 | 36.7 | 144,401 |
| 62 single test | - | 13.6 | 14 | 38.3 | 157,753 |
| 63 single test | - | 13.8 | 14.7 | 39.8 | 169,896 |
| 64 single test | - | 13.8 | 15.2 | 41.4 | 181,158 |
| 65 single test | - | 13.7 | 15.6 | 43.1 | 191,658 |
| 66 single test | - | 13.3 | 15.7 | 44.7 | 200,704 |
| 67 single test | - | 12.7 | 15.6 | 46.4 | 208,283 |
| 68 single test | - | 12.1 | 15.4 | 48.1 | 213,560 |
| 69 single test | - | 11.3 | 15 | 49.8 | 217,594 |

PCM= prostate cancer mortality reduction; LYs_gain = life-years gain

ǂ Life years gain and costs are 3.5% discounted

# Compared to no screening
