# Supplementary material for: Assessment of harms, benefits, and cost‐effectiveness of prostate cancer screening: A micro‐simulation study of 230 scenarios
Source: Cancer Med. 2020 Aug 19;9(20):7742–50. doi: 10.1002/cam4.3395 (PMC7571827; doi:10.1002/cam4.3395)
Supplement: Supplementary file 3 — Supplementary Material [file CAM4-9-7742-s003.docx]

**Assessment of Harms, Benefits and Cost-effectiveness of Prostate Cancer Screening: A Micro-Simulation Study of 230 Scenarios**

**Supplementary materials**

**Calibration and validation of the model**

We used the model of Heijnsdijk et al. 2015 as a base for this study^15^. Model parameters for the natural history of prostate cancer (including transition probabilities and mean dwelling time), stage dependent test sensitivities and lead time dependent cure probability were estimated based on the following data:

-Baseline incidence and stage distribution in 1991-1993 in the Netherlands,

-ERSPC Rotterdam trial data up to July 2004 (screen results until 2006) for both arms of the trial,

-Baseline incidence in Sweden in 1990, and

-ERSPC results of Göteborg up to end of 2004.

During the calibration, the baseline incidence and incidence in control arm, detection rate in first and subsequent screens, interval cancers, clinical T-stage distribution, metastatic state and biopsy Gleason score distribution were used. In addition, the model was calibrated to the incidence of the Dutch population in 1992–2002.

**Model updates in the current study**

Because the previous model didn’t account for men younger than 55 years, we recalibrated the model to Dutch incidence between 1989 to 2013 by 5-year age categories from age 50 to age 75. Parameters for the hazards of onset of a pre-clinical prostate tumor and clinical prostate cancer detection were calibrated to these data. During the calibration, for the period 1989 to 1992 we used no screening, and after 1992 we used an estimate of increasing opportunistic PSA screening in the Netherlands, varying from 16% (within five years) for age group 50-55% to 41% (within five years) for age group 65-75% (based on CBS.nl).

Following this, we projected the incidence of prostate cancer (to check the performance of the calibration) over the period from 1989 to 2013 by 5 years age category from age 50 to 75 (with no screening scenario from year 1989 to 1992, and with opportunistic screening from year 1993 to 2013 (Appendix Figure 1). We also predicted the prostate cancer mortality over the same period, age categories and screening strategies, and compared with the observed prostate cancer mortality among the Dutch population to validate our model, Appendix Figure 2. (**Note:** the model was not calibrated to the Dutch prostate cancer mortality data).
